# The Mediating Role of Alcohol Use Between Adverse Childhood Experiences and Delinquency Among Youth in the Legal System

**DOI:** 10.3390/ijerph23010095

**Published:** 2026-01-09

**Authors:** Akemi E. Mii, Johanna B. Folk, Brandon D. L. Marshall, Kathleen Kemp, Sophia Garcia-Meza, Marina Tolou-Shams

**Affiliations:** 1 Department of Psychiatry and Behavioral Sciences, University of California, San Francisco, CA 94110, USA; 2 Department of Epidemiology, Brown University School of Public Health, Providence, RI 02903, USA; 3 Department of Psychiatry and Human Behavior, Warren Alpert Medical School, Brown University, Providence, RI 02903, USA

**Keywords:** adverse childhood experiences, juvenile legal, alcohol use, delinquency, petition

## Abstract

Rates of alcohol use and exposure to adverse childhood experiences (ACEs) are elevated among youth in the legal system (YILS) compared to their non-legally involved peers. Exposure to ACEs has been associated with later alcohol use and delinquency, and YILS often engage in delinquent behavior while under the influence of alcohol. The associations between ACEs, alcohol use, and delinquency among YILS are complex and multidirectional; research has yet to explore how these experiences and behaviors influence each other over time, or whether they differ based upon the reason for legal system involvement (status or delinquent petition). This study examined whether YILS’ ACEs were prospectively associated with self-reported delinquent behaviors, and whether self-reported recent alcohol use was an explanatory mechanism for this association. Multigroup mediation analyses were utilized to examine if these pathways differed based on youth’s court petition type. Results indicated that YILS report high rates of ACEs. Frequency of recent alcohol use mediated the associations between ACEs and subsequent delinquency for youth with a delinquent, but not status, petition. Concurrent assessment of trauma exposure and alcohol use when youth first enter the legal system is imperative to inform early intervention needs to reduce the likelihood of continued system involvement.

## 1. Introduction

Youth in the legal system (YILS) commonly report high rates of adverse childhood experiences (ACEs), such as abuse, neglect, or household dysfunction. ACEs have a dose–response relationship with delinquency, suggesting that the more ACEs one experiences, the greater the likelihood of engagement in delinquent behaviors. Given that substance use is also a salient predictor of delinquency [1] and the high rates of alcohol use among YILS [2], understanding how alcohol use may influence the association between ACEs and delinquency is warranted to further inform and tailor intervention.

YILS have particularly high rates of alcohol use and using alcohol has been associated with continued engagement in delinquent behaviors, which can impact continued legal system involvement [2,3]. Given the high rates of ACE exposure and alcohol use among YILS, it is possible that YILS may use alcohol to cope with thoughts and feelings associated with an adverse experience. To our knowledge, however, the longitudinal associations between ACEs, alcohol use, and engagement in delinquent behaviors among YILS have not been examined. This study explores how ACEs and alcohol use prospectively impact engagement in self-reported delinquent behavior to inform effective prevention and early intervention efforts that promote positive outcomes for youth. This is particularly needed as the existing empirically supported assessments and interventions focused on substance use among YILS are not specific to alcohol use [4].

### 1.1. Youth in the United States Legal System

Youth may enter the United States juvenile legal system due to either status or delinquent petitions. A status offense refers to acts that are only criminalized for minors (e.g., truancy, alcohol use), whereas a delinquent offense refers to acts that are criminalized regardless of the person’s age (e.g., assault, burglary). In 2022, the United States juvenile courts processed 62,000 status petitions and 549,500 delinquent petitions [5]. Of note, much of the research examining YILS has focused on youth with delinquent petitions. This may be a result of issues including: (1) the belief that delinquent offenses are more “severe” than status offenses, (2) differences in how states classify and process status petitions (e.g., reclassification of status offense behaviors that place youth in the child welfare rather than juvenile legal system [5], and (3) efforts made to reduce consequences of status-level petitions, which aligns with the deinstitutionalization of the Juvenile Justice and Delinquency Prevention Act [6]. While status offenses are only criminalized due to the youth’s age, they can have serious legal and psychosocial consequences for youth. Thus, additional research is needed to better understand youth with status petitions to determine if they have unique needs compared to youth with delinquent petitions.

### 1.2. Adverse Childhood Experiences

The 10 standard ACEs include experiences of abuse (physical, sexual, and emotional), neglect (physical and emotional), and household dysfunction (caregiver substance use, caregiver mental health concern, divorce or separation, caregiver incarceration, and witnessing domestic violence) and these ACEs have been associated with a range of negative mental, behavioral, and physical health outcomes [7,8]. However, the standard ACEs framework was developed based on predominately white, middle-to-upper class adults with college-level education and commercial insurance, and critiques of this framework have focused on the generalizability of these findings to diverse youth populations that are not represented in the original study. An expanded ACEs framework was developed among a sample of adults living in an urban city with varying ethno-racial identities, education statuses, and socioeconomic statuses [9]. The expanded ACEs framework includes five types of community or social adversity: witnessing community violence, experiencing discrimination, living in an unsafe neighborhood, experiencing bullying, and living in foster care. Among this sample, 64% of adults endorsed at least one expanded ACE [9]. Prior research suggests that YILS have experienced multiple expanded ACEs by the first time they have contact with the legal system [10].

Research on ACE exposure among YILS has focused predominately on the standard ACEs, revealing that YILS endorse high rates of exposure compared to community samples of youth [11,12]. YILS are 13 times more likely than peers to report a history of standard ACEs [11]. A meta-analysis of 423,000 YILS found prevalence rates for individual ACEs ranging from 13.7% (household member with mental health concerns) to 52.3% (exposure to domestic violence) [13]. Less is known about the prevalence and impact of expanded ACEs among YILS. Folk and colleagues [10] found that among this sample of youth at their first contact with the legal system, YILS reported experiencing, on average, three standard and two expanded ACEs; however, the number of expanded ACEs youth endorsed did not predict any additional mental health needs over and above the standard ACEs. Nonetheless, ACEs among YILS are associated with increased mental and behavioral health concerns, which in turn is related to greater needs for mental and behavioral health services and risk for continued system involvement [14,15]. Further research is needed to clarify if and how expanded ACEs may differentially impact YILS mental health and substance use to inform treatment needs.

While much of the existing research has focused on the association between ACEs and recidivism among YILS [16], growing evidence suggests ACEs can be associated with engagement in delinquent behaviors more broadly [17,18,19]. Research has found that specific types of ACEs (e.g., child sexual abuse, neglect) and the number of ACEs are associated with youth’s engagement in delinquent behavior [19,20]. Of note, these studies are largely correlational; thus, causation cannot be implied. Multiple studies have found that higher ACE scores are associated with more frequent engagement in delinquent behaviors [17,18,21]. Additionally, research examining ACEs categories (e.g., abuse, neglect, household dysfunction) has found that certain types of ACEs may drive the associations with mental health and substance use outcomes. Specifically, Folk and colleagues [12] found that abuse ACEs predicted subsequent alcohol use. Thus, it is critical to examine both the number and categories of ACEs to understand mechanisms driving this association to inform prevention and intervention efforts that reduce delinquency and continued system involvement for these youth.

### 1.3. Alcohol Use

Alcohol use and its consequences are a significant concern during adolescence, with recent estimates approximating that 757,000 United States adolescents (ages 12 to 17) were diagnosed with a recent alcohol use disorder (AUD) in 2023 [22]. Not all alcohol use in adolescence leads to AUD, but it is important to understand how youth use alcohol and when use becomes a concern. A recent meta-analysis synthesizing over 100 studies of community adults, though not specifically legally involved, found that cumulative ACE exposure was moderately associated with substance use, including problematic alcohol use [*OR* = 1.812 (95% *CI* 1.606, 2.044)] and heavy alcohol use [*OR* = 1.537 (95% *CI* 1.344, 1.758)] [23]. These estimates suggest that individuals with higher ACE exposure are approximately 1.5–2 times more likely to engage in harmful alcohol use compared to those with fewer ACEs.

YILS use alcohol at high rates and at early ages [2,24]. Specifically, Prinz and Kerns [24] found that approximately 60% of their sample tried alcohol by the age of 13 and a substantial proportion of YILS who used alcohol by 13 years of age reported drinking alcohol at least several times per month. Early initiation of alcohol use has been associated with a variety of chronic cognitive, neurobiological, and socioemotional consequences [25,26]. Additionally, in a sample of over 1800 YILS, 81% of youth reported using alcohol in the past year and 45% of the youth that reported using alcohol had used it at least 10+ times during that year [2]. Given the early initiation, frequency of alcohol use, and associated consequences, additional research is needed to better understand the drivers of alcohol use among YILS.

Coping with the effects of ACEs is one possible explanation. In a community-based sample of youth, the number of standard and expanded ACEs were positively associated with alcohol use [27]. Further, ACE exposure has been associated with an earlier age of alcohol use onset [28,29]. However, these findings have not been consistently documented among YILS, with some research suggesting that ACEs are associated with more frequent substance use or substance use problems [30,31], while others find a negative association [32,33]. Most research to date has examined ACEs and broad substance use, without a specific focus on alcohol use.

Finally, alcohol use is positively associated with engagement in delinquent behavior and recidivism among YILS, and the association between these may be reciprocal [3,34]. Most research on the association between substance use and delinquency among YILS has focused on recidivism. Among over 1800 arrested youth, a diagnosis of alcohol and/or drug use disorder was associated with the initiation, escalation, and continuation of offending behaviors, and the odds of re-offending approximately doubled for those who used substances [35]. A recent study among detained youth found that substance use was associated with a higher risk of reoffending, such that risk increased by 6.7% with each additional ACE [36]. Further, YILS who used alcohol and/or drugs in the past six months presented with more risk factors across several domains for recidivism (e.g., school, relationships, skills) compared to YILS who did not [37]. Given high rates of alcohol use among YILS and the associations between alcohol, delinquency, and legal involvement, it is evident that research assessing alcohol use among YILS is warranted.

### 1.4. Purpose of the Current Study

The associations between ACEs, alcohol use, and delinquency among YILS are complex and multidirectional. Yet, most of the research with YILS in this area has focused on the 10 standard ACEs, broad substance use, and rates of recidivism as the primary outcome of interest. Further, research understanding how ACEs, alcohol use, and delinquency influence each other longitudinally has yet to be examined, particularly among youth at their first contact with the legal system. Interventions for youth at their first contact with the legal system can provide more effective services to reduce the likelihood of continued system involvement, though these may need to be tailored for youth, based on their reason for involvement in the system. It is therefore warranted to explore how these associations may differ among youth with status vs. delinquent petitions at their first court contact.

This study aimed to address these gaps by longitudinally examining how YILS’ experiences of standard and expanded ACEs impacted their subsequent engagement in delinquent behaviors. We hypothesized that this association would be mediated by the frequency of recent alcohol use. Further, this study employed multigroup mediation analyses to examine if these pathways differed based on initial petition type.

## 2. Materials and Methods

### 2.1. Participants and Procedure

Youth and caregivers were recruited for participation in a 2 year longitudinal study from a large family court located in the Northeastern United States. This statewide family court provides oversight of the juvenile services department, which handles all juvenile legal petitions filed in the state. Once a status and/or delinquent petition is opened for a youth under the age of 18 years, the intake services department conducts a preliminary investigation to assess if there is sufficient evidence to bring the case to court, and if so, what actions need to be taken to ensure the safety of the public and youth. Cases that are not brought to the formal court calendar are diverted in various different ways, such as referrals to court and community-based diversion programs, including specialty or collaborative courts. Baseline data were collected between June 2014 and July 2016. Youth were eligible if they met the following criteria: (1) they resided in the community, (2) they were between 12 and 18 years old; (3) they had a first-time, open status and/or delinquent petition filed in the past 30 days; (4) they had no prior history of court involvement; (5) they had no significant cognitive impairment that would preclude providing informed assent; and (6) they had a caregiver living in the same household with them for at least six months prior to enrollment who was also willing to participate. The sample and procedures of this study are consistent with the parent study [38]. Study flyers were distributed to families with their court date notification letters. At the first court appointment, research assistants met with youth and caregivers to discuss the study and complete a private, separate screening with families interested in participating. Eligible and interested youth and caregivers met with research staff outside of the court setting (e.g., at home, private community space, research lab) to provide assent and consent for study participation. Girls were intentionally oversampled to allow for examination of gender differences, and youth with open delinquent and status petitions were equally sampled. Over 90% of participating youth with a status petition were referred through truancy court. Assessments were administered via a tablet with an audio-assisted computerized assessment in English and Spanish (for caregivers only). Additional study procedures are described in prior manuscripts [12,38]. All study procedures were approved by the Institutional Review Board at the University of California, San Francisco.

The parent study included 401 youth–caregiver dyads who were consented and followed for 24 months, completing assessments approximately every 4 months (for a total of seven timepoints: see [38] for additional details). All youth (*n* = 313) and caregivers (*n* = 324) who participated in the first 4-month follow-up assessment were offered the opportunity to separately consent/assent for the youth to complete additional (one-time) assessment regarding past potentially traumatic experiences; 273 youth completed these supplementary measures. Of the 273 youth, 271 completed the 12-month follow-up assessment and 262 responded to questions regarding expanded ACEs. Thus, the 262 youth–caregiver dyads comprise the analytic sample for the current study. Youth demographics for this sub-study largely mirror those of the full study [38].

Given that abuse and neglect ACEs data were only collected at one timepoint (as part of an additional study assessment), these were only available for a subset of the full sample. Independent sample t-tests and chi-square tests were conducted to assess differences between youth with and without complete ACEs data. Youth with missing data on abuse ACEs (χ2 (1, *N* = 368) = 5.393, *p* = 0.020) and neglect ACEs (χ2 (1, *N* = 368) = 4.361, *p* = 0.037) were more likely to identify as male. No significant differences were found based on age, Latine identity, prior alcohol use at baseline assessment, 8 month alcohol use, or delinquency scores at baseline and 12 month assessments.

The baseline and 4-month timepoints were selected based on when ACEs were first assessed in the full study; the 12 month follow-up primary outcome timepoint was selected for the current study to extend prior findings with this sample [10,12]. The sample and procedures for the current study mirror those described in depth in a prior manuscript, which also examined ACEs in this same longitudinal cohort [38].

### 2.2. Measures

Demographics. Youth and caregivers reported on their demographic characteristics (e.g., gender, age, race, and ethnicity) at the baseline assessment.

Standard ACEs. Consistent with prior manuscripts utilizing this sample that assess ACEs [10,12], the 10 standard ACEs [39,40] were assessed through a series of instruments administered at the baseline assessment and the first 4-month follow-up (see Table 1 for measures and scoring). ACE exposure reflected lifetime experiences up to and including 4 months post-baseline assessment. When both the youth and caregiver reports were available, an affirmative response by either was considered endorsement of the ACE. Scores were prorated if youth had missing data for no more than two standard ACEs. ACEs were examined categorically (i.e., abuse ACEs, neglect ACEs, household dysfunction ACEs, and expanded ACEs). The total number of standard and expanded ACEs was also examined, consistent with prior manuscripts [10,12] and other prior research among YILS (e.g., [15,41,42]). Standard ACE categories were assessed as follows:

Abuse. Physical, emotional, and sexual abuse were assessed at 4-month follow-up assessment via the Childhood Trauma Questionnaire—Short Form (CTQ-SF; [43]). The scale has good internal consistency, ω = 0.80, 95% *CI* [0.65, 0.88]. Based on recommended a priori cutoff scores (low to moderate = 9–12; moderate to severe = 13–15; severe to extreme = >15), youth with scores of nine or greater on any subscale were considered to have experienced that specific type of abuse [44]. One item from the Traumatic Life Events Inventory (TLE; [45]) was also used to identify youth with exposure to sexual abuse (e.g., rape, attempted rape, made to perform any type of sexual act through force or threat of harm). If a youth reported experiencing any of these events, they were considered to have experienced sexual abuse.

Neglect. Physical and emotional neglect were assessed at the 4-month follow-up on the CTQ-SF [43]. Youth with scores of nine or greater on any subscale were considered to have experienced that specific type of neglect.

Household dysfunction. Household dysfunction ACEs included the following: caregiver substance use, caregiver mental illness, exposure to domestic violence, caregiver separation or divorce, and caregiver incarceration. Questions answered on the Adolescent Risk Behavior Assessment (ARBA; [46]) and an adapted version for parents (Parent Risk Behavior Assessment; PRBA), as well as the Parent Arrest and Treatment History questionnaire (PATH; [38]) were utilized to assess exposure to household dysfunction (see prior manuscript for more information [38]). The PRBA and the PATH [38] were completed by caregivers at the baseline and 4-month follow-up timepoints to assess for lifetime exposure to household dysfunction occurring from birth to the 4-month follow-up assessment. Similarly, the ARBA was completed by adolescents at the baseline and 4-month follow-up timepoints to assess for lifetime exposure to household dysfunction occurring from birth to the 4-month follow-up assessment. See Table 1 for the specific items utilized to gather information for each particular household dysfunction ACE.

Expanded ACEs. Expanded ACEs were assessed through a series of instruments administered at the baseline and 4-month follow-up timepoints to gather lifetime exposure to these events, as discussed in a prior manuscript utilizing this sample [10]. Table 1 also provides information on expanded ACE measurement. Each individual ACE was coded as 1 = yes or 0 = no, accounting for endorsement of that expanded ACE at the baseline and/or 4-month follow-up assessments (possible range = 0–5). Scores were prorated if youth had missing data for no more than one expanded ACE.

Witnessing violence was assessed at the 4-month follow-up, using five items from the TLE [45].

Discrimination based on race or ethnicity was assessed at the baseline assessment, using the 11-item Everyday Discrimination Scale (EDS; [47]). The scale has excellent internal reliability, ω = 0.93, 95% *CI* [0.91, 0.94]. Youth self-reported their experiences with discrimination in their day-to-day life on 10 items (scale from 1 = almost every day to 6 = never). Items were reverse scored and summed to create a total score; higher values reflected more frequent experiences with discrimination.

Adverse neighborhood experience was assessed at the baseline assessment, using the 6-item neighborhood disadvantage subscale from the Neighborhood Environment Scale (NES; [48,49]). Items are rated as 1 = true or 0 = false and then summed to yield an overall score. The neighborhood disadvantage subscale at the baseline assessment had good reliability, ω = 0.87, 95% *CI* [0.84, 0.89].

Experience with bullying was assessed at the baseline assessment, using a single item.

Placement in foster care was assessed at the baseline and the four-month follow-up assessments using two items. Therapeutic foster care was defined to families as “where foster parents have been trained to provide care”.

Alcohol use. Youth self-reported frequency of recent (past 120 days) alcohol use on the ARBA at the baseline and 8-month follow-up assessments [46]. Youth’s lifetime history of alcohol use reported at the baseline assessment was dichotomized (i.e., used alcohol or has not used alcohol) and provided for descriptive information on the sample. The primary alcohol use outcome is frequency of use (i.e., number of days) over the prior 120 days, reported at the 8-month follow-up assessment (henceforth referred to as recent alcohol use). It is of note that this variable was positively skewed and leptokurtic; thus, it was log-transformed for analysis. The transformed variable had acceptable skewness and kurtosis and was used for subsequent analyses.

Delinquency. Youth self-reported engagement in 23 types of delinquent acts (recent delinquency) through the National Youth Survey (NYS; [50]) during the prior 120 days at the baseline and at the 12-month follow-up assessments. The number of acts endorsed was summed to create a self-report general delinquency score. Despite good internal reliability, (α = 0.79), two items were excluded from Cronbach’s alpha estimation, due to zero or one participant reporting those acts, and they did not contribute any variance (i.e., forcing someone to have sex and committing robbery). This variable was positively skewed and leptokurtic, so it was log-transformed for analysis; the transformed variable had acceptable skewness and kurtosis and was used for subsequent analyses.

### 2.3. Data Analytic Plan

**Figure 1:**
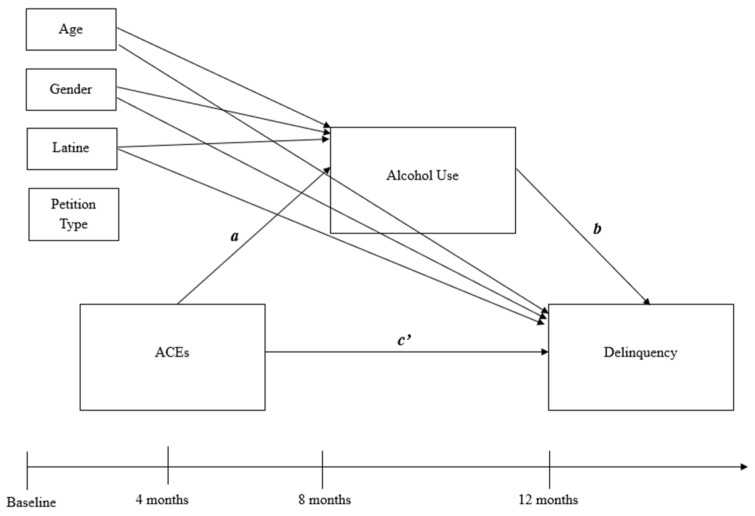
Conceptual mediation model with pathways and assessment timepoints. Note. Conceptual model depicts which variables were collected at which assessment timepoints. ACEs were collected at the baseline and 4-month assessment timepoints. Path *a* refers to the direct effect of ACEs on alcohol use. Path *b* refers to the direct effect of alcohol use on delinquency. Path *c’* refers to the direct effect of ACEs on delinquency when statistically controlling for the effect of alcohol use.

Descriptive and bivariate analyses were conducted using the Statistical Package for the Social Sciences version 29. Descriptive statistics were examined to determine if the data met the basic assumptions of path analysis. Categories of ACEs that were significantly correlated with recent delinquency (reported at the 12-month follow-up) or recent alcohol use (reported at the 8-month follow-up) were included in the multigroup mediation models (see Figure 1 for the conceptual model). Next, multigroup mediation models were conducted using Mplus software 8.11 [51] with maximum likelihood estimation with robust standard errors. Multigroup mediation models were tested across two groups (based on petition type: status vs. delinquent) to assess if the pathways between ACEs and recent delinquency were mediated by recent alcohol use, and if these pathways significantly differed based on youth’s petition type. All multigroup mediation analyses co-varied youth’s age, gender, and ethnicity (i.e., Latine vs. non-Latine), so that group differences in the mediation models were estimated after statistically controlling for these demographic characteristics. Consistent with prior analyses related to ACEs using this analytic sample [10,12], ethnicity was dichotomized due to small sample sizes within the Black, multiracial, and other non-Latine groups. Of note, analyses examining formally tested interactions of demographic characteristics are preliminary, as this study was not specifically designed or powered for these analyses. Originally, baseline alcohol use and delinquency were included as covariates in the models; however, the multigroup mediation models could not be computed due to low variability, particularly among youth with status petitions, thus precluding their inclusion in the models.

The data met the basic assumption of path analyses and models were just identified; thus, global model fit statistics are not informative. Cases with missing data were retained if at least one path in the model was observed. The covariance coverage for these analyses ranged from 0.808 to 1.00. See Table 2 for sample sizes across measures and timepoints. Missing data in this sample was relatively low and comparable across groups. Among youth with status petitions, missing data were observed in 8-month frequency of alcohol use (<3%) and 12-month recent delinquency (<16%). For youth with delinquent petitions, missing data were observed in 8-month frequency of alcohol use (<10%) and <10% on 12-month recent delinquency. Baseline predictors and covariates (ACEs, age, gender, ethnicity) had complete data. Full information maximum likelihood was used to address the missing data.

Lastly, regarding the number of zeros in the delinquency outcome variable, we were not able to use other count-data in the multigroup mediation analyses, as they made the models unstable. These models cannot be computed with both the mediator and outcome as count variables in a mediation framework. Instead, we conducted sensitivity analyses in which we tested a, b, and c paths in each group separately, using Poisson and negative binomial models. These models indicated the same pattern of results as our log-transformed data (see Supplementary Materials for sensitivity analyses). Log transformation of delinquency and alcohol use allowed for improved distribution properties and stable estimations with robust maximum likelihood (MLR), which provides robust standard errors and parameter estimates under non-normality and missingness.

## 3. Results

### 3.1. Descriptive Statistics and Bivariate Associations

Youth (*N* = 262) were 14.54 years old on average (*SD* = 1.59) at the time of enrollment. Males and females were fairly split among the sample, with 51.9% identifying as male. Youth self-identified as 49.8% White, 17.3% Multiracial, 16.5% Black, and 16.5% “Other”. Youth identifying as Latine comprised 42.5% of the sample. Youth with a status petition constituted 51.1% of the sample (*n* = 134) and 48.9% of youth had a delinquent petition (*n* = 128). Table 3 provides additional youth demographics and adverse childhood experiences (ACEs) endorsed, based on petition type.

Independent sample t-tests and chi-square difference tests were conducted to examine if there were differences in the key variables of interest among the samples, based on petition type (see Table 3). Youth with a delinquent petition were significantly older than youth with a status petition, *t*(259.46) = −3.17, *p* = 0.002. Youth identifying as female were more likely to have a status petition, while youth identifying as male were more likely to have a delinquent petition, *χ*^2^ (1, *N* = 260) = 5.61, *p* = 0.018. Petition type did not differ based on racial or Latine identity.

Youth with a delinquent petition had higher delinquency scores at the baseline assessment compared to youth with a status petition, *t*(230.36) = −4.33, *p* < 0.001. There were no significant differences in recent delinquency scores at the 12-month follow-up based on petition type, or in ACEs, lifetime alcohol use, or 8-month recent alcohol use.

Correlations to examine associations between categories of ACEs (i.e., abuse, neglect, household dysfunction, expanded, and total ACEs) and key outcomes revealed that among youth with a status petition, only neglect was positively correlated with recent delinquency, *r*(97) = 0.21, *p* = 0.042. For youth with a delinquent petition, abuse ACEs were positively correlated with frequency of recent alcohol use and recent delinquency scores, *r*(94) = 0.34, *p* < 0.001 and *r*(94) = 0.29, *p* = 0.005, respectively. Total ACEs were positively correlated with frequency of recent alcohol use, *r*(99) = 0.26, *p* = 0.008, and frequency of recent alcohol use was positively associated with recent delinquency, *r*(93) = 0.54, *p* < 0.001.

### 3.2. Mediational Models

#### 3.2.1. Total ACEs, Alcohol Use, and Delinquency by Petition Type

For youth with a status petition, there were no significant associations between total ACEs, frequency of recent alcohol use, or recent delinquency scores (see Table 4). Age was positively associated with 8-month follow-up frequency of recent alcohol use (standardized coefficient = 0.297, *SE* = 0.070, *p* < 0.001). Youth who identified as non-Latine endorsed more frequent recent alcohol use at the 8-month follow-up (standardized coefficient = −0.196, *SE* = 0.075, *p* = 0.009). Gender was not associated with 8-month follow-up frequency of recent alcohol use. Age, gender, and Latine identity were not associated with recent delinquency at the 12-month follow-up.

**Figure 2:**
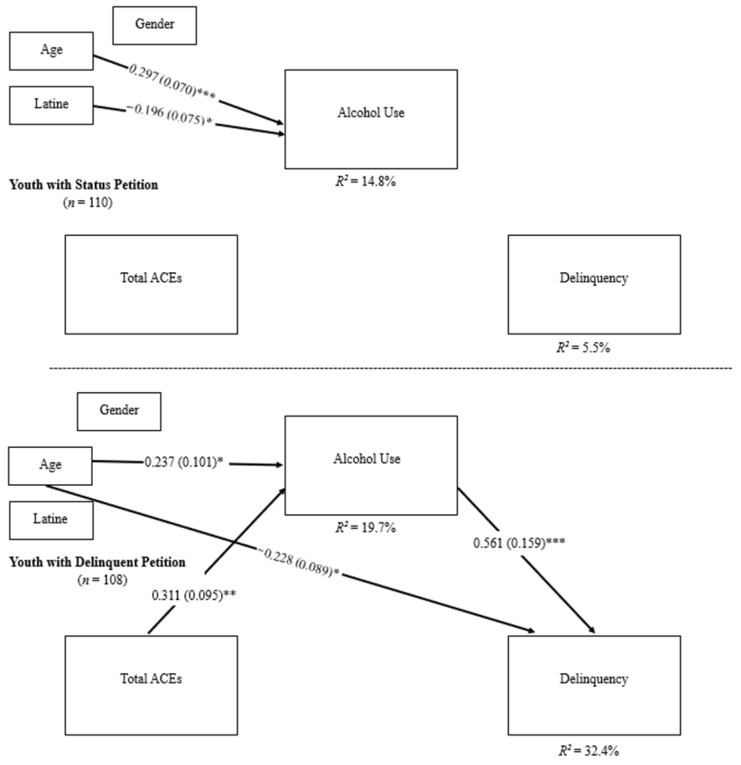
Multigroup mediation analysis for total ACEs. Path estimates were compared across groups based on petition type and found a significant difference between total ACEs on alcohol use path and alcohol use on delinquency path. A significant difference in the indirect effect of alcohol use on total ACEs and delinquency was found between the two groups. Standardized coefficients are reported in the figure. * *p* < 0.05, ** *p* < 0.005, and *** *p* < 0.001.

For youth with a delinquent petition, there was a direct effect of total ACEs on frequency of recent alcohol use at the 8-month follow-up (standardized coefficient = 0.311, *SE* = 0.095, *p* = 0.001). There was also a direct effect of frequency of recent alcohol use on recent delinquency scores at the 12-month follow-up (standardized coefficient = 0.561, *SE* = 0.159, *p* < 0.001). However, there was no direct effect of total ACEs on 12-month recent delinquency when accounting for 8-month follow-up recent alcohol use (standardized coefficient = 0.051, *SE* = 0.087, *p* > 0.05). Age was positively associated with frequency of recent alcohol use at the 8-month follow-up (standardized coefficient = 0.237, *SE* = 0.101, *p* = 0.020) and inversely associated with recent delinquency scores at the 12-month follow-up (standardized coefficient = −0.228, *SE* = 0.089, *p* = 0.010). Gender and Latine identity were not associated with either frequency of recent alcohol use or recent delinquency scores, *p* > 0.05 (see Figure 2 and Table 4).

In comparing youth based on petition type, significant differences emerged in the associations between total ACEs and frequency of recent alcohol use (Δ*B* = −0.100, *SE* = 0.048, *p* = 0.038) and frequency of recent alcohol use and recent delinquency (Δ*B* = −0.274, *SE* = 0.094, *p* = 0.003). Additionally, a significant difference in the indirect effect of frequency of recent alcohol use at the 8-month follow-up on total ACEs and recent delinquency at the 12-month follow-up was found between the two groups, (ΔIndirect Effect = −0.031, *SE* = 0.014, *p* = 0.030), suggesting that frequency of recent alcohol use mediated the association between total ACEs and recent delinquency among youth with delinquent but not status petitions (see Table 4 and Figure 2).

#### 3.2.2. Abuse ACEs, Alcohol Use, and Delinquency by Petition Type

For youth with a status petition, there was a direct effect of abuse ACEs on recent delinquency scores at the 12-month follow-up (standardized coefficient = 0.225, *SE* = 0.110, *p* = 0.040). There was no direct effect of abuse ACEs on frequency of recent alcohol use, or of frequency of recent alcohol use on recent delinquency scores. Age was positively associated with frequency of recent alcohol use at the 8-month follow-up (standardized coefficient = 0.307, *SE* = 0.069, *p* < 0.001). Males (standardized coefficient = −0.176, *SE* = 0.088, *p* = 0.044) and youth who identified as non-Latine (standardized coefficient = −0.211, *SE* = 0.073, *p* = 0.004) reported more frequent recent alcohol use. Age, gender, and Latine identity were not associated with recent delinquency scores, *p* > 0.05.

**Figure 3:**
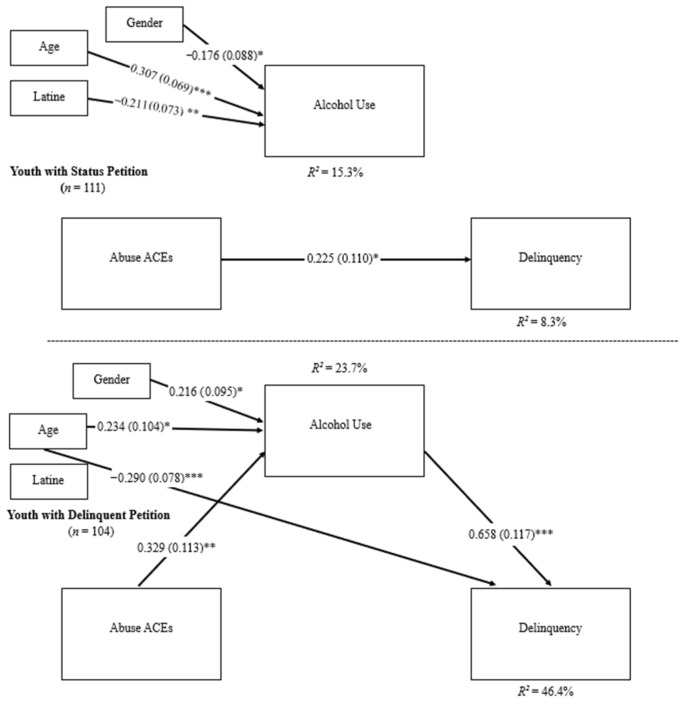
Multigroup mediation analysis for abuse ACEs. Path estimates were compared across groups based on petition type and found a significant difference between abuse ACEs on alcohol use and alcohol use on delinquency paths. A significant difference in the indirect effect of alcohol use on abuse ACEs and delinquency was found between the two groups. Standardized coefficients are reported in the figure. * *p* < 0.05, ** *p* < 0.005, and *** *p* < 0.001.

For youth with a delinquent petition, there were direct effects of abuse ACEs on frequency of recent alcohol use (standardized coefficient = 0.329, *SE* = 0.113, *p* = 0.004) and of frequency of recent alcohol use on recent delinquency scores (standardized coefficient = 0.658, *SE* = 0.117, *p* < 0.001). However, there was no direct effect of abuse ACEs on recent delinquency scores at the 12-month follow-up when accounting for the influence of recent alcohol use reported at the 8-month follow-up (standardized coefficient = 0.134, *SE* = 0.079, *p* > 0.05). Age was positively associated with a higher frequency of recent alcohol use (standardized coefficient = −0.234, *SE* = 0.104, *p* = 0.025). Identifying as female was associated with a higher frequency of recent alcohol use (standardized coefficient = 0.216, *SE* = 0.095, *p* = 0.023). Conversely, younger age was associated with higher recent delinquency scores (standardized coefficient = −0.290, *SE* = 0.078, *p* < 0.001). Gender and Latine identity were not associated with either frequency of recent alcohol use or recent delinquency scores, *p* > 0.05 (see Table 4 and Figure 3).

In comparing youth based on petition type, significant differences emerged in the association of recent alcohol use to recent delinquency (ΔB = −0.342, *SE* = 0.075, *p* < 0.001). Additionally, a significant difference in the indirect effect of frequency of recent alcohol use on abuse ACEs and recent delinquency was found between the two groups (ΔIndirect Effect = −0.107, *SE* = 0.052, *p* = 0.039), suggesting that the frequency of recent alcohol use reported at the 8-month follow-up mediated the association between abuse ACEs and recent delinquency at the 12-month follow-up among youth with delinquent petitions (see Table 4 and Figure 3).

#### 3.2.3. Neglect ACEs, Alcohol Use, and Delinquency by Petition Type

For youth with a status petition, there was a direct effect of neglect ACEs on recent delinquency scores at the 12-month follow-up (standardized coefficient = 0.243, *SE* = 0.112, *p* = 0.031). There were no direct effects of neglect ACEs on frequency of recent alcohol use or frequency of recent alcohol use on recent delinquency. Age was positively associated with higher frequency of recent alcohol use at the 8-month follow-up (standardized coefficient = 0.218, *SE* = 0.084, *p* = 0.010). Identifying as non-Latine was associated with a greater frequency of recent alcohol use (standardized coefficient = −0.224, *SE* = 0.068, *p* = 0.001). Gender was not associated with frequency of recent alcohol use. Age, gender, and Latine identity were not associated with recent delinquency scores, *p* > 0.05.

**Figure 4:**
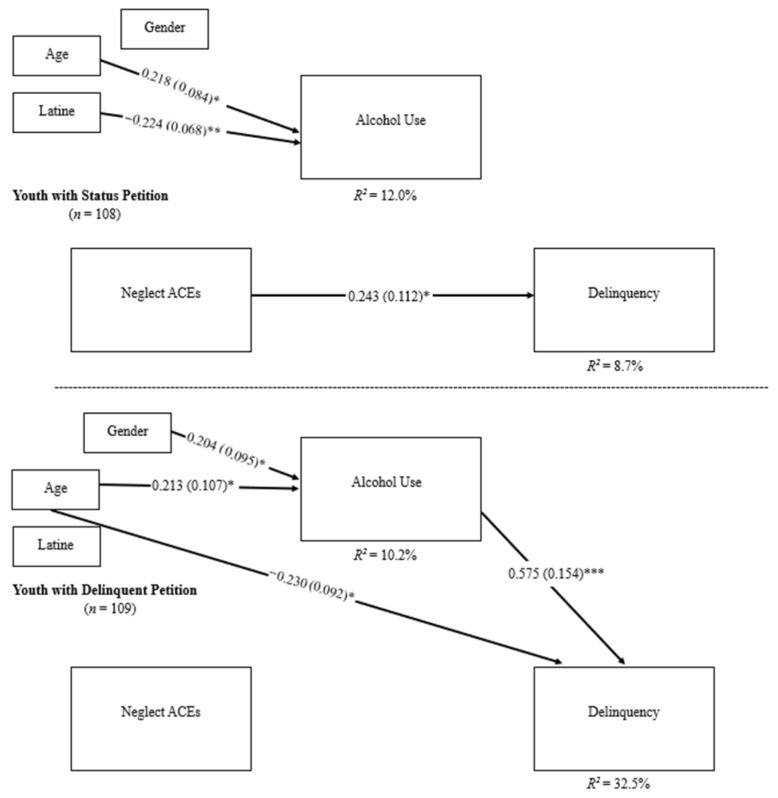
Multigroup mediation analysis for neglect ACEs. Path estimates were compared across groups based on petition type and found no significant difference of neglect ACEs on alcohol use. There was a significant difference across groups of alcohol use on delinquency. There was no significant difference in the indirect effect of alcohol use on neglect ACEs and delinquency found between the two groups. Standardized coefficients are reported in the figure. * *p* < 0.05, ** *p* < 0.005, and *** *p* < 0.001.

For youth with a delinquent petition, frequency of recent alcohol use had a direct effect on recent delinquency (standardized coefficient = 0.575, *SE* = 0.154, *p* < 0.001). However, there was no direct effect of neglect ACEs on frequency of recent alcohol use at the 8-month follow-up nor neglect ACEs on recent delinquency at the 12-month follow-up. Age was positively associated with a higher frequency of recent alcohol use (standardized coefficient = 0.213, *SE* = 0.107, *p* = 0.046). Identifying as female was associated with a higher frequency of recent alcohol use (standardized coefficient = 0.204, *SE* = 0.095, *p* = 0.032). Conversely, a younger age was associated with higher scores of recent delinquency (standardized coefficient = −0.230, *SE* = 0.092, *p* = 0.012. Gender was not associated with recent delinquency. Latine identity was not associated with either frequency of recent alcohol use or recent delinquency scores, *p* > 0.05 (see Table 4 and Figure 4).

In comparing youth based on petition type, the only significant difference that emerged was between the frequency of recent alcohol use to recent delinquency pathway (ΔB = −0.315, *SE* = 0.088, *p* < 0.001). There was no significant difference in the indirect effect of frequency of recent alcohol use on neglect ACEs and recent delinquency found between the two groups (see Table 4 and Figure 4).

## 4. Discussion

The current study adds to the growing body of research examining associations between ACEs, alcohol use, and delinquency across time for YILS. Consistent with prior research, YILS reported high rates of exposure to ACEs; 95.2% of the youth endorsed at least one standard or expanded ACE, including 48% having experienced some form of abuse. There were no differences in the amount of ACE exposure among youth based on petition type, but differential pathways between ACEs, alcohol use, and subsequent delinquency were observed. Findings underscore opportunities to provide tailored trauma-informed screening and interventions for youth at their first contact with the legal system to appropriately address their behavioral health needs.

Research with YILS examining ACEs and delinquency has largely focused on recidivism, yet this only represents a subset of youth engaging in delinquent behavior, as recidivism is inconsistently defined in the literature and there are systemic and systematic biases in who has additional recorded contact with the legal system [52]. This study examined self-reported delinquent behavior more broadly, which allows for a more thorough understanding of youth who are engaging in delinquent behaviors but may not have contact with the legal system as a result.

At the bivariate level, experiencing abuse, neglect, or total ACEs was prospectively associated with self-reported delinquent behavior. Thus, we examined these specific ACE categories within multigroup mediation models to better understand how alcohol use influences this association. A compact summary table is provided to highlight key significant and non-significant pathways (see Table 5). Across groups (i.e., status, delinquent) and models (i.e., total ACEs, abuse ACEs, and neglect ACEs), findings highlighted statistically significant indirect effects, such as that frequency of recent alcohol use statistically mediated the associations between ACEs, specifically total and abuse, and recent delinquency among youth with delinquent petitions within the constraints of the specified models. However, frequency of recent alcohol use did not mediate the associations between ACEs and recent delinquency for youth with a status petition. In the delinquency petition group, the standardized path from ACEs to alcohol use (β ≈ 0.32) was broadly consistent with, and somewhat larger than, the small-to-moderate associations reported in recent meta-analytic work linking cumulative ACE exposure to problematic or heavy alcohol use (pooled *OR*s ≈ 1.5–1.8). The path from alcohol use to delinquency (β ≈ 0.65) was large, yielding an indirect effect of approximately β ≈ 0.21, indicating a medium-sized mediated association. These findings build upon existing mixed results, revealing an association between ACEs and substance use among YILS [53], and suggest there are different contributors to delinquency for youth who enter the juvenile legal system due to acts that are illicit solely due to being a minor (status), or those that are illicit regardless of age (delinquent). Alcohol use may reflect both coping mechanisms for thoughts and feelings associated with adversity and as a behavior that increases youth involvement in contexts where delinquency is more likely to occur. However, these interpretations should be viewed as plausible exploratory hypotheses, rather than definitive causal mechanisms. Given these statistically mediated pathways for YILS with delinquent petitions, assessing and, if necessary, treating alcohol use may serve as an important intervention need.

Demographic differences in who entered the court due to delinquent vs. status petitions were also revealed. Consistent with official juvenile court statistics showing that females comprise a small proportion of those receiving delinquent petitions (e.g., 28% out of the 549,500 delinquent cases processed in 2022 [5], youth with status petitions in this sample were more likely to identify as female and be younger than those with delinquent petitions. Although pathways to delinquency differ for girls and boys, with adversity playing a salient role [54,55], it is important to highlight that these interactional models were not sufficiently powered and thus our findings should be interpreted with caution. Our findings revealed that females with delinquent petitions who experienced abuse or neglect reported more frequent recent alcohol use, highlighting the gendered ways in which trauma and substance use can intersect with delinquent behavior. However, as noted, our models were not sufficiently powered; thus, more formal testing of these gendered differences is needed, particularly as most research on YILS has been conducted with samples of boys and interventions are often developed and tested with boys in mind. Girls may be more likely to use substances, such as alcohol, as a coping response to interpersonal and relational adversity, whereas boys may be more likely to engage in behaviorally driven or externalizing pathways to delinquency. Extending such findings risks overlooking critical gender-specific mechanisms and needs. Future research should prioritize gender-responsive approaches that recognize girls’ distinct trauma histories, coping strategies, and pathways to legal system involvement. This includes testing whether existing interventions are equally effective for girls and developing and adapting treatments that explicitly account for gender.

Our findings also revealed that non-Latine youth with status petitions reported more frequent alcohol use. Research indicates that cultural norms and beliefs among Latine youth may be protective against alcohol use; for example, strong positive family relations and greater parental restrictions that limit opportunities to drink have been associated with decreased frequency of alcohol use among Latine youth [56,57]. Taken together, these findings demonstrate that youths’ intersecting identities are an important consideration when youth enter the legal system.

## 5. Strengths, Limitations, and Future Directions

The strengths of this study include the prospective design (including assessment of the exposure, mediators, and outcomes at different timepoints), use of empirically validated assessments, multi-informant approach, examination of both standard and expanded ACEs, and examination of youth petition type. The inclusion of petition type as a key analytic focus represents a unique contribution, extending prior research on ACEs and substance use among YILS to better capture heterogeneity within this population.

As these were secondary data analyses from a parent study that did not explicitly focus on trauma, there are several limitations regarding ACE measurement. Specifically, this study did not utilize a single ACE questionnaire. Instead, data gathered through various assessments were triangulated to determine exposure to ACEs. Additionally, most ACEs were measured at the baseline and 4-month follow-up. However, one of the expanded ACEs (i.e., adverse neighborhood experience) was only assessed at the baseline assessment. Thus, there may have been exposure to adverse neighborhood experiences between the baseline and 4-month follow-up assessment timepoints that were not gathered. Second, although the longitudinal study design strengthens our temporal ordering, the multigroup mediation models reflect statistical mediation, rather than definitive causal mechanisms. For example, there was a low variance in scores for a variety of measures. Specifically, youth self-reported frequency of recent alcohol use and recent engagement in delinquent behavior scores were log-transformed for path analyses due to the low variability. Similarly, it was not possible to control for alcohol use and delinquency scores at the baseline assessment in the models due to this low variance. Thus, the indirect pathways should be interpreted as exploratory and associational, rather than causal. Relatedly, there may be other uncontrolled factors that could confound the exposure–outcome, exposure–mediator, and mediator–outcome associations modeled in this study. While this study focused specifically on alcohol use, it is possible that YILS who use multiple substances (e.g., alcohol and other drugs) have differential pathways from ACEs to delinquency. Future research is needed to further examine these associations among YILS, using other substances or engaging in polysubstance use.

Additionally, research examining ACEs among YILS has predominately been deficit-based. Thus, research understanding positive childhood experience and malleable resiliency factors are important in informing treatment. Due to small subgroup sample sizes, it was not possible to explore more nuanced analyses of differences between specific demographic populations in these associations and our findings warrant further testing. Future research should prioritize considering how intersecting identities influence early identification and screening practices to ensure that risk is not assessed through a one-size-fits-all lens. Lastly, these data are also part of a larger study that recruited from one family court in the Northeastern United States and may not be representative of all youth at their first contact with the juvenile legal system. Although the age, gender, and ethno-racial identities of this sample are largely consistent with those reported in the 2022 Juvenile Court Statistics [5], it is important to note that the context in which these proceedings occur may not be largely representative. Specifically, juveniles in this state are processed through a large family court that processes petitions for the majority of youth in a single small state.

## 6. Practical Implications

Exposure to ACEs should not be used solely to justify a need for trauma treatment, as many youth who experience ACEs do not exhibit trauma symptoms. However, adopting trauma-informed approaches to screening and identifying treatment needs when working with YILS is imperative. For example, courts and community agencies could implement brief, validated trauma and alcohol screening tools, such as the UCLA PTSD Reaction Index for trauma symptoms and the Alcohol Use Disorders Identification Test (AUDIT) for alcohol use. These tools could be incorporated into existing intake procedures, including entrance to probation, diversion programs, and family court intake. Thus, if youth’s screeners indicate concerns for trauma symptoms or substance use, this would inform appropriate referral needs. Further, the usage of these more comprehensive screening approaches in the juvenile legal system and emphasis on providing staff and other appropriate legal staff with psychoeducation regarding the impact of ACEs and other forms of trauma can promote YILS well-being.

Findings also indicate that girls in the legal system may be more likely to use alcohol in response to ACEs exposure. While most research examining adolescent substance use among YILS is with boys, this study’s findings suggest that more research in understanding substance use among legally involved girls is warranted, as girls’ experiences of adversity have been associated with substance use and girls disproportionately experience interpersonal adversity [58,59]. Gender-responsive substance use treatment that incorporates a relational approach has been associated with reduced cannabis use, as well as reduced traumatic stress and other psychiatric symptoms for legally involved girls [60].

These findings also highlight potential policy implications. Our findings suggest that YILS have experienced high rates of ACEs prior to their first contact with the legal system, and alcohol use can influence the path from ACEs to delinquency for some youth. Policy approaches that focus primarily on punitive sanctions risk overlooking underlying trauma and behavioral health needs. Rather than treating delinquent behavior as an isolated incident, adopting a trauma-informed and holistic approach to addressing YILS behaviors may improve youth outcomes and reduce continued engagement in delinquent behavior. Specifically, this approach could be integrated across various levels of the legal system, including at diversion, probation intake, and family court. This may look like establishing standardized screening assessments that consider youth’s history of adversity and mental and behavioral health symptoms, and how these may be associated with the youth’s engagement in delinquent behavior. Further, the incorporation of these standardized screenings may improve referral pathways to ensure that youth are receiving appropriate treatment.

## 7. Conclusions

Youth at their first contact with the legal system experience high rates of adversity regardless of the legal reason for system contact. For youth with a delinquent petition, frequency of recent alcohol use mediated the association between experiences of abuse and subsequent delinquency. However, alcohol use did not mediate this association for youth with a status petition. Findings indicate the importance of screening for alcohol use and targeted, trauma-informed substance use interventions for youth with a delinquent petition.

## Figures and Tables

**Table 1:** Adverse childhood experiences (ACEs) measurement.

	Domain	Reporter	Instrument	Items	Timepoint
**Standard ACEs**	Abuse				
	Physical	Y	CTQ-SF	Physical Abuse subscale ≥ 9	4 mo
	Emotional	Y	CTQ-SF	Emotional Abuse subscale ≥ 9	4 mo
	Sexual	Y	CTQ-SF; TLE	Sexual Abuse subscale ≥ 9 TLE: Endorsed “Happened to me” to “Sexual assault”	4 mo
	Neglect				
	Physical	Y	CTQ-SF	Physical Neglect subscale ≥ 9	4 mo
	Emotional	Y	CTQ-SF	Emotional Neglect subscale ≥ 9	4 mo
	Household Dysfunction				
	Substance Use	Y; CG	CTQ-SF; PRBA	CTQ-SF: “My parents were too drunk or high to take care of the family.” PRBA: (1) “Have you been told by a doctor you have a diagnosis of a substance use disorder?” (2) “Have you been in treatment for drug and/or alcohol problems?”	BL + 4 mo
	Mental Health	CG	PRBA	(1) “Have you been told by a doctor you have a psychiatric diagnosis?” (2) “Have you been in treatment for mental health difficulties?”	BL + 4 mo
	Violence	Y	ARBA	“Have you seen your parent get pushed, slapped, hit, punched, or beat up by another parent, or their boyfriend or girlfriend?”	BL + 4 mo
	Separation	Y; CG	Demo	Y: Endorse “step-parent” to “What is your relationship to the parent or caregiver who is participating with you?” CG: “What is your current marital status?”	BL + 4 mo
	Incarceration	CG	PATH	“Have you ever been incarcerated?”	BL + 4 mo
**Expanded ACEs**	Witnessing Violence	Y	TLE	Endorsed “Witnessed” to (1) physical assault, (2) assault with a weapon, (3) sexual assault, (4) sudden violent death, (5) serious injury, harm, or death you caused to someone else	4 mo
	Discrimination	Y	EDS	Endorsed “A few times a year or more” to any item and due to their ancestor or national origin, race, or shade of skin color	BL
	Neighborhood	Y	NES	6 items summed. Higher scores = higher levels of neighborhood disadvantage	BL
	Bullying	Y	Demo	“Have you ever been bullied?”	BL
	Foster Care	Y; CG	Demo	(1) “Have you/your child been removed from the home by DCYF or another state child welfare agency?” (2) “Have you/your child been in therapeutic foster care?”	BL + 4 mo

Note. BL = baseline; Y = youth; and CG = caregiver, mo = month. All instrument acronyms are reported in the abbreviation table at the end of the manuscript.

**Table 2:** Assessment completion across timepoints by petition type.

Petition Type	Baseline	4-Month ACEs					8-Month Alcohol	12-Month Delinquency
		Abuse	Neglect	HD	Expanded	Total ACEs		
Status	134	122	118	89	134	120	120	106
Delinquent	128	107	112	102	128	111	103	111
Total Sample	262	229	230	191	262	231	223	217

**Table 3:** Sample characteristics based on petition type.

	Total *N* = 262	Status *N* = 134	Delinquent *N* = 128	Difference
Demographics				
Age	*M* = 14.53 *SD* = 1.59	*M* = 14.23 *SD* = 1.64	*M* = 14.84 *SD* = 1.49	*t* = −3.17 p *=* 0.002
Gender	51.9% male	55.3% female	59.4% male	*χ*^2^ = 5.61 *p* = 0.018
Latine	42.5% Latine	37.4% Latine	47.7% Latine	*χ*^2^ = 2.79 *p* = 0.095
American Indian	10.6%	12.2%	8.9%	*χ*^2^ = 0.75 *p* = 0.386
Asian	2.0%	3.1%	0.8%	*χ*^2^ = 1.67 *p* = 0.196
Black	16.5%	16.0%	16.9%	*χ*^2^ = 0.038 *p* = 0.846
Native Hawaiian or Other Pacific Islander	0.8%	0.0%	1.6%	*χ*^2^ = 2.13 *p* = 0.144
White	49.8%	51.1%	48.4%	*χ*^2^ = 0.194 *p* = 0.660
Mixed or Multiracial	17.3%	17.6%	16.9%	*χ*^2^ = 0.017 *p* = 0.896
Other	16.5%	15.3%	17.7%	*χ*^2^ = 0.284 *p* = 0.594
ACEs				
Abuse ACEs	48.0% endorsed	46.7% endorsed	49.5% endorsed	*t* = −0.95 *p* = 0.345
Neglect ACEs	57.0% endorsed	56.8% endorsed	57.1% endorsed	*t* = −0.73 *p* = 0.467
Household Dysfunction ACEs	56.0% endorsed	52.8% endorsed	58.8% endorsed	*t* = −0.73 *p* = 0.469
Expanded ACEs	83.2% endorsed	84.3% endorsed	82.0% endorsed	*t* = 1.78 *p* = 0.073
Total ACEs	95.2% endorsed	96.7% endorsed	93.7% endorsed	*t* = 0.317 *p* = 0.752
Outcomes				
Baseline Lifetime Alcohol Use	22.9% used prior	19.4% used prior	26.6% used prior	*χ*^2^ = 1.90 *p* = 0.168
8-Month Frequency of Recent Alcohol Use	*M* = 2.19 *SD* = 10.02	*M* = 0.98 *SD* = 3.55	*M* = 3.60 *SD* = 14.15	*t* = −1.83 *p* = 0.07
Baseline Delinquency	*M* = 2.33 *SD* = 2.75	*M* = 1.63 *SD* = 2.23	*M* = 3.07 *SD* = 3.04	*t* = −4.33 *p* < 0.001
12-Month Recent Delinquency	*M* = 0.36 *SD* = 0.99	*M* = 0.25 *SD* = 0.85	*M* = 0.47 *SD* = 1.10	*t* = −1.59 *p* = 0.113

Note. In this study, youth gender was collected via a self-report item with the options “female” or “male”. We acknowledge that “female” and “male” are appropriately classified as sex categories rather than gender identities. We use the terms “female” and “male” descriptively to reflect categories in our data because these are what participants self-selected, though acknowledge that conflation of gender and sex terminology limits the accurate representation of gender identity. Throughout the manuscript, “female” and “male” are used when referencing other studies reporting sex, and “girls” and “boys” when referencing other studies reporting gender. Much of the literature among YILS, and youth more broadly, uses gender and sex terminology interchangeably and often in binary forms, which limits our knowledge about gender differences in relation to the topics of focus.

**Table 4:** Multigroup mediation analyses.

Path	Status Petition		Delinquent Petition		
	β (SE)	B (SE)	β (SE)	B (SE)	Difference ΔB
Total ACEs					
Total ACEs → Alcohol	0.095 (0.076)	0.022 (0.018)	0.311 (0.095)	0.122 (0.045)	−0.199 (0.048) *p* = 0.038
Age → Alcohol	0.297 (0.070)	0.113 (0.034)	0.237 (0.101)	−161 (0.065)	-
Gender → Alcohol	−0.166 (0.092)	−0.196 (0.105)	0.178 (0.094)	0.371 (0.197)	-
Latine → Alcohol	−0.196 (0.075)	−0.245 (0.108)	−0.014 (0.087)	−0.028 (0.177)	-
Alcohol → Delinquency	−0.041 (0.098)	−0.021 (0.051)	0.561 (0.159)	0.253 (0.078)	−0.274 (0.094) *p* = 0.003
Total ACEs → Delinquency	0.188 (0.162)	0.022 (0.019)	0.051 (0.087)	0.009 (0.015)	0.013 (0.025) *p* = 0.582
Age → Delinquency	−0.110 (0.113)	−0.022 (0.022)	−0.228 (0.089)	−0.070 (0.030)	-
Gender → Delinquency	−0.073 (0.111)	−0.044 (0.070)	−0.040 (0.090)	−0.038 (0.086)	-
Latine → Delinquency	−0.081 (0.123)	−0.052 (0.080)	−0.042 (0.089)	−0.039 (0.081)	-
Abuse ACEs					
Abuse → Alcohol	0.066 (0.100)	0.047 (0.075)	0.329 (0.113)	0.336 (0.140)	−0.288 (0.159) *p* = 0.070
Age → Alcohol	0.307 (0.069)	0.119 (0.034)	0.234 (0.104)	0.154 (0.065)	-
Gender → Alcohol	−0.176 (0.088)	−0.209 (0.104)	0.216 (0.095)	0.437 (0.194)	-
Latine → Alcohol	−0.211 (0.073)	−0.262 (0.106)	−0.007 (0.082)	−0.014 (0.163)	-
Alcohol → Delinquency	−0.042 (0.088)	−0.026 (0.054)	0.658 (0.117)	0.316 (0.052)	−0.342 (0.075) *p* < 0.001
Abuse → Delinquency	0.225 (0.110)	0.098 (0.048)	0.134 (0.079)	0.065 (0.040)	0.032 (0.063) *p* = 0.604
Age → Delinquency	−0.149 (0.092)	−0.035 (0.021)	−0.290 (0.078)	−0.091 (0.027)	-
Gender → Delinquency	−0.091 (0.107)	−0.065 (0.076)	−0.143 (0.074)	−0.139 (0.078)	-
Latine → Delinquency	−0.116 (0.095)	−0.087 (0.078)	−0.066 (0.083)	−0.064 (0.079)	-
Neglect ACEs					
Neglect → Alcohol	0.129 (0.079)	0.115 (0.073)	0.085 (0.091)	0.105 (0.117)	0.010 (0.137) *p* = 0.942
Age → Alcohol	0.218 (0.084)	0.093 (0.037)	0.213 (0.107)	0.144 (0.068)	-
Gender → Alcohol	−0.111 (0.092)	−0.146 (0.115)	0.204 (0.095)	0.424 (0.196)	-
Latine → Alcohol	−0.224 (0.068)	−0.309 (0.116)	−0.023 (0.091)	−0.046 (0.185)	-
Alcohol → Delinquency	−0.105 (0.075)	−0.057 (0.044)	0.575 (0.154)	0.258 (0.077)	−0.315 (0.088) *p* < 0.001
Neglect → Delinquency	0.243 (0.112)	0.117 (0.063)	0.043 (0.079)	0.024 (0.044)	0.093 (0.077) *p* = 0.226
Age → Delinquency	−0.097 (0.085)	−0.022 (0.020)	−0.230 (0.092)	−0.070 (0.031)	-
Gender → Delinquency	−0.016 (0.096)	−0.012 (0.069)	−0.043 (0.088)	−0.040 (0.085)	-
Latine → Delinquency	−0.151 (0.092)	−0.113 (0.076)	−0.049 (0.090)	−0.045 (0.082)	-

**Table 5:** Summary table for key pathways in multigroup mediation models.

Model	Petition Type	ACE → Alcohol	Alcohol → Delinquency	ACE → Delinquency	Indirect Effect
Total ACEs	Status	ns (β = 0.095)	ns (β = −0.041)	ns (β = 0.188)	ns
	Delinquent	sig (β = 0.311)	sig (β = 0.561)	ns (β = 0.051)	sig
Abuse ACEs	Status	ns (β = 0.066)	ns (β = −0.042)	sig (β = 0.225)	ns
	Delinquent	sig (β = 0.329)	sig (β = 0.658)	ns (β = 0.134)	sig
Neglect ACEs	Status	ns (β = 0.129)	ns (β = −0.105)	sig (β = 0.243)	ns
	Delinquent	ns (β = 0.085)	sig (β = 0.575)	ns (β = 0.043)	ns

Note. ns = non-significant; sig = significant.

## Data Availability

The data that support the findings of this study are not publicly available due to the sensitive nature of the information and to protect participant privacy and confidentiality.

## Supplementary Materials

The following supporting information can be downloaded at: https://www.mdpi.com/article/10.3390/ijerph23010095/s1, Table S1: Sensitivity Analyses Using Count Models for a-, b-, and c′-Paths Stratified by Petition Type.

## Abbreviations

The following abbreviations are used in this manuscript:

ACEs	Adverse Childhood Experiences
YILS	Youth in the Legal System
AUD	Alcohol Use Disorder
CTQ-SF	Childhood Trauma Questionnaire—Short Form
TLE	Traumatic Life Events
ARBA	Adolescent Risk Behavior Assessment
PRBA	Parent Risk Behavior Assessment
PATH	Parent Arrest and Treatment History
EDS	Everyday Discrimination Scale
NES	Neighborhood Environment Scale
NYS	National Youth Survey
