# Supplementary material for: The Mediating Role of Alcohol Use Between Adverse Childhood Experiences and Delinquency Among Youth in the Legal System
Source: Int J Environ Res Public Health. 2026 Jan 9;23(1):95. doi: 10.3390/ijerph23010095 (PMC12840722; doi:10.3390/ijerph23010095)
Supplement: Supplementary file 1 [file ijerph-23-00095-s001.zip › ijerph-3991485-supplementary.pdf]

Supplementary Materials

Sensitivity Analyses Using Count Models for a-, b-, and c′-Paths Stratified by Petition Type

Path Tested	Model	B	SE	*p*	IRR
	Status Petition				
ACEs → Alcohol (a-path)	Poisson	0.130	0.054	.016	1.139
	Zero Inflated Poisson	0.147	0.078	.058	1.158
	Negative Binomial	0.278	0.146	.057	1.32
Alcohol → Delinquency (b-path)	Poisson	-0.119	0.184	.518	0.888
	Zero Inflated Poisson	-0.147	0.156	.344	0.863
	Negative Binomial	-0.129	0.173	.455	0.879
ACEs → Delinquency (c′-path)	Poisson	0.172	0.148	.245	1.188
	Zero Inflated Poisson	0.092	.155	.552	1.096
	Negative Binomial	0.160	0.117	.173	1.174
	Delinquent Petition				
ACEs → Alcohol (a-path)	Poisson	0.342	0.109	.002	1.408
	Zero Inflated Poisson	0.188	0.083	.024	1.207
	Negative Binomial	0.383	0.126	.002	1.467
Alcohol → Delinquency (b-path)	Poisson	0.070	0.015	< .001	1.073
	Zero Inflated Poisson	0.042	0.021	0.041	1.043
	Negative Binomial	0.070	0.013	< .001	1.073
ACEs → Delinquency (c′-path)	Poisson	0.021	0.1072	.776	1.021
	Zero Inflated Poisson	0.035	0.060	.557	1.036
	Negative Binomial	0.062	0.066	.346	1.064

Note. B = unstandardized coefficient. SE = standard error. IRR = Incidence Rate Ratios (IRRs = e^B) are reported instead. IRR > 1 = increased outcome rate; IRR < 1 = decreased outcome rate.

Poisson and Negative Binomial models were estimated separately based on petition type to evaluate robustness of the a-, b-, and c′-paths relative to the primary log-transformed mediation model.

Sensitivity analyses using Poisson, Zero Inflated Poisson, and negative binomial models yielded the same substantive conclusions as the primary log-transformed models. Among youth with delinquent petitions, ACEs were

associated with increased frequency of alcohol use and alcohol use was associated with delinquency, while ACEs were not directly associated with delinquency when alcohol was included. No paths were statistically significant among youth with status petitions. These findings indicate that the results are robust to alternative distributional specifications.
